# Supplementary material for: A whole-animal phenotypic drug screen identifies suppressors of atherogenic lipoproteins
Source: eLife. 2026 Aug 27;14:RP105314. doi: 10.7554/eLife.105314 (PMC13521566; doi:10.7554/eLife.105314)
Supplement: Supplementary file 2 [file elife-105314-code1.zip › JHDL_primary_result/Unknown/Unknown_JHDL_4_016_016_2019_05_10_11_29.pdf]

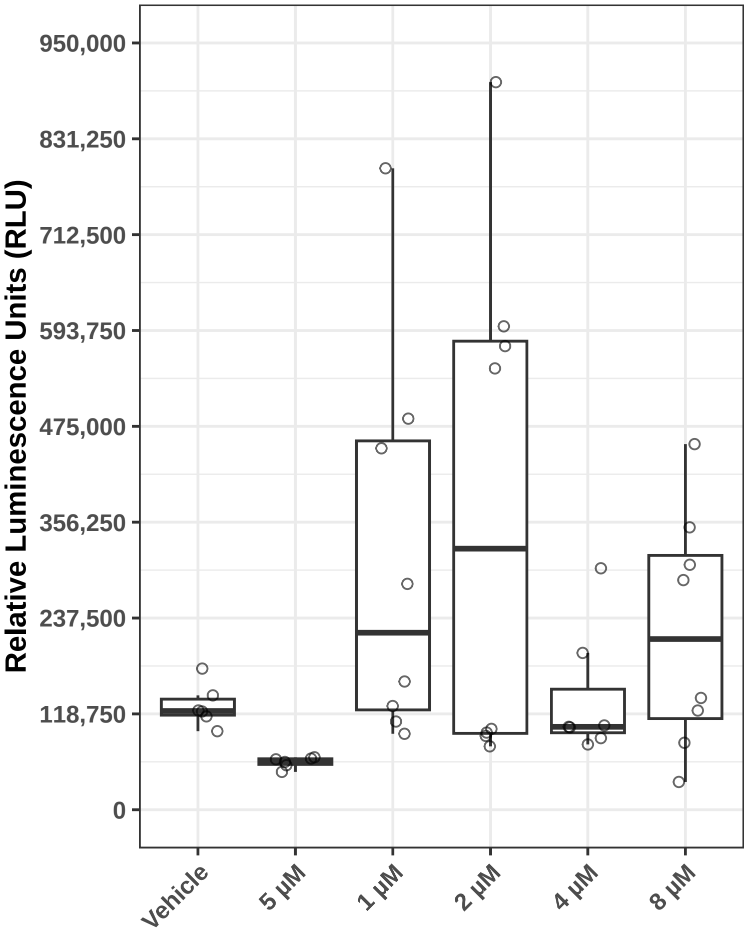

Relative Luminescence Units (RLU)
950,000
831,250
712,500
593,750
475,000
356,250
237,500
118,750
0
Vehicle
5 µM
1 µM
2 µM
4 µM
8 µM
